# A literature review on work transitioning of youth with disabilities into competitive employment

**DOI:** 10.4102/ajod.v6i0.298

**Published:** 2017-08-29

**Authors:** Madri Engelbrecht, Lynn Shaw, Lana van Niekerk

**Affiliations:** 1 Division of Occupational Therapy, University of Stellenbosch, South Africa; 2 School of Occupational Therapy, Faculty of Health Professions, Dalhousie University, Canada; 3 Division of Occupational Therapy, Tygerberg Campus, University of Stellenbosch, South Africa

## Abstract

**Background:**

The marginalisation of youth with disabilities from employment opportunities is evident from literature in as far as they form part of the larger groups ‘people with disabilities’ and ‘youth’. A focused view of programmes that assist youth with disabilities into employment has not been presented, despite the worldwide crisis of youth unemployment.

**Aim:**

This review aimed to identify evidence on work transition programmes that are effective in assisting people with disabilities into open labour market (competitive) employment, as well as to highlight gaps in knowledge to inform future research on this topic.

**Methods:**

Literature and policy on programmes that support such transitions were considered, firstly from a global perspective and then with a view from developing countries. The SALSA (Search, Appraisal, Synthesis and Analysis) framework was used to source and analyse information from a diverse set of documents. Various online databases were searched for research papers published between 1990 and 2016, and websites were searched for reports pertaining to this topic.

**Results:**

Ninety-nine documents were selected to inform the review, out of an identified 259 scientific journal articles, policy documents, acts, organisational reports and book chapters.

**Conclusion:**

A synthesis of findings was presented in a narrative that reflects the themes of youth with disabilities and employment in the world, work transition endeavours in the developing world and a specific focus on this group in South Africa. The review revealed a gap in knowledge and evidence pertaining to youth with disabilities and employment, highlighting these as research foci, and emphasising the need for youth-focused research that generates knowledge about disability and transitions into the labour force.

## Introduction

Across the world, youth have been identified as a vulnerable group who experiences low levels of employment. In 2014, 75 million out of the 200 million unemployed people worldwide were youth (International Labour Organisation [ILO] 2014). Youth development has also become a critical priority for South Africa. Here, youth is defined as people between the ages of 14 and 35, with the upper age limit so high because of historical imbalances that were created by the apartheid regime (National Youth Development Agency 2015). This age group comprises a disturbing 71% of the unemployed population (Statistics South Africa 2012) and is among the worst affected by the 2008/2009 recession (Department of Labour 2012).

A minority group of youth, namely youth with disabilities, has not been prioritised by governments in creating access to employment for them. Although South African policy identified youth with disabilities as a priority target group a decade ago, the government has, for example, opted not to apply a quota system in labour legislation that facilitates employment of people with disabilities, even though such a strategy is regarded a viable method to increase employment (ILO 2015).

Information about youth with disabilities is scarce. One reason may be that statistics about this group are reported as part of general disability statistics. For instance, current employment statistics in South Africa reflect 1.2% of the workforce as people with disabilities (Department of Labour 2015), with no indication of the proportion of youth with disabilities. Others have noted that youth with disabilities have largely been ignored in development efforts for young people, with more research focused on adults than youth with disabilities (Lindsay, McDougall, Menna-Dack, Sanford & Adams 2015).

This review of the literature and policies was set against the above backdrop, which reflects the absence of a plan for youth with disabilities in relation to employment.

## Methods of the literature search

The first author of this paper conducted a review of programmes that support the transition of youth with disabilities into competitive employment. Her objective was to identify evidence about programmes that are effective and knowledge gaps about youth with disabilities in relation to employment, which could inform future research directions in this field. A further aim was to develop an understanding about local and international disability discourses that might inform increased labour participation opportunities for youth with disabilities. The primary research question that led the review was ‘What knowledge and evidence contribute to the successful transition of youth with disabilities into employment from international and local (South African) perspectives or literature?’ Given the need to first focus broadly and then to examine literature and evidence from a local perspective, a systematic approach to an integrative review process was followed (Whittemore & Knafl 2005), by applying the SALSA (Search, Appraisal, Synthesis and Analysis) framework (Grant & Booth 2009). This framework supports flexibility and unique processes rather than adopting a specific literature review type. It was used to conduct an organised review by sourcing and analysing information on the complex and challenging social issue of youth with disabilities and transitions into competitive employment.

### Search

The authors acknowledged that different perspectives were needed to identify, analyse and explain how evidence from an international perspective might be used to inform and enhance South African policy, research directions and the application of evidence in practice. The sourcing of diverse types of literature to produce information is consistent with conducting an integrative review of evidence to improve health practices (Whittemore & Knafl 2005). This approach was thus adopted for the integration of information that might lead to the improvement of employment practices for youth with disabilities. Sub-questions were developed and key words identified to guide the search strategies (Table 1). These were used to find information from research papers, policy documents and legislation or institutional reports. Research databases CINAHL, MEDLINE, PsycINFO, Elsevier, Wiley Online Library, SAGE Publications and ArticleFirst were searched for research papers published from 1990 until March 2016. Local and international government, disability organisations and research institutions websites were searched for relevant reports and documents. Websites included those of the International Labour Organisation (ILO), World Health Organisation (WHO), Disabled People South Africa (DPSA), the South African Departments of Labour, Social Development, and Health, and the Human Sciences Research Council (HSRC). The search process resulted in a total of 259 documents being identified, including 164 scientific journal articles, 92 policy documents, acts, organisational reports, and five books or chapters from books.

**TABLE 1:** Search strategies and key words.

Sub-questions to guide search strategies	Key words used
What types of knowledge exist in the international and local literature on youth with disabilities and transitions into employment?	Youth with disabilities Youth unemployment People with disabilities and unemployment Developing countries Open labour market
What are the strategies or models that support how youth with disabilities enter into employment in the international and local literature?	Youth with disabilities Unemployment Transition programmes School-to-work Work transition strategies
What research has been done on employment outcomes for youth with disabilities and transitions into employment?	Disability & poverty Disability & inequality Welfare to workfare Labour market policy Youth with disabilities & employment People with disabilities & unemployment
Is there evidence of successful work transition programmes?	Youth transitioning into work Youth with disabilities & employment Transitioning youth with disabilities
What, if any, are the international standards for transitioning youth with disabilities into employment?	Youth employment transition programmes Youth with disabilities School-to-work

*Source:* Authors’ own work

### Appraisal

Ninety-nine articles and documents were selected to inform this review, after those that reflected duplication of information, or did not present current evidence, or diverged from the topic of youth with disabilities, or reported on forms of employment not included in this review, were excluded. To establish the fit and relevance of the literature (Arksey & O’Malley 2005), abstracts or executive summaries were read and key words were highlighted (Table 1). Next, the potential of the document to contribute information to answer the sub-questions in Table 1 was reviewed.

### Synthesis

**FIGURE 1:**
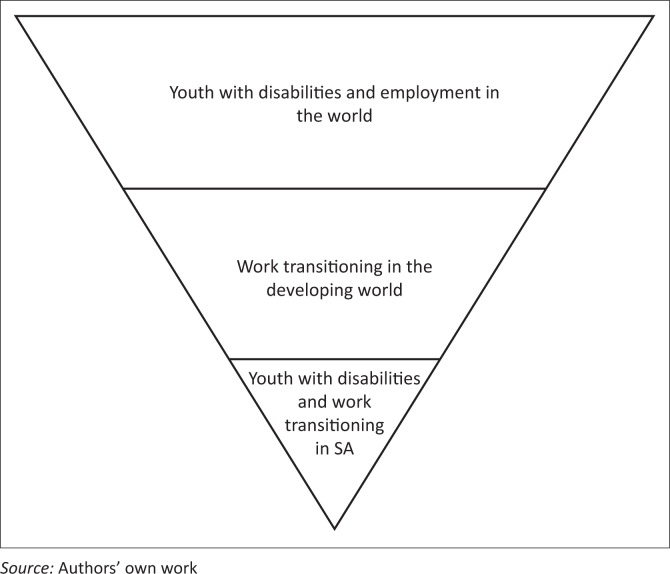
Themes used to extract information from literature.

The guiding sub-questions as well as deep reading and reflection upon the documents were used to generate a framework for the extraction and synthesis of information. The framework included the contexts (global and local) and levels of evidence (i.e. macro-economic, policy and operational levels) that organised and supported the extraction of information, and the subsequent integration thereof. The themes in Figure 1 were used to obtain information about global and local platforms and to guide an explanation of the implementation of approaches in countries outside South Africa, as well as in South Africa, on youth with disabilities transitioning into work, and to identify the research that is needed to enhance practice and policy.

### Analysis

An integrative approach was used to interpret and combine the context of system influences with evidence, policy and reports on programme outcomes aimed at achieving employment inclusion of youth with disabilities. A holistic synthesis of the information on each theme was drafted and critically appraised to identify what knowledge is missing, and what research is needed to enhance inclusion and participation of youth with disabilities. Recommendations for areas of research that will inform policy and practice were identified. The results of the analysis were shared with two researchers for coherence with the state of knowledge globally (international advisor) and locally (local supervisor). A narrative summary of the findings was produced.

## Youth with disabilities and employment in the world

Youth with disabilities’ marginal position in terms of employment has been recognised and described by researchers (Groce 2004; Lindsay, Hartman & Fellin 2015). Youth with disabilities are often unemployed, under-employed or earn less than their non-disabled counterparts (Groce 2004). They are often the last to be hired and the first to be retrenched or fired, or hired for jobs that require little training and have few opportunities for development. Even when they are well educated, youth with disabilities take longer to find a position, have less job security and less prospect of advancement than their non-disabled peers with similar levels of education (Groce 2004). These disadvantages are compounded, with fewer youth with disabilities working or looking for work than non-disabled youth (Lindsay, Hartman & Fellin 2015).

Youth with disabilities’ outlook for entering into employment is further limited by a basic lack of work and employment preparation. A critical examination of the broad disability and development literature in low-income countries (for example Bangladesh, Cambodia and Kenya) reports that the disadvantages of youth with disabilities in employment start with the denial of opportunities to participate as a child (Parnes et al. 2009). Access to early, primary and secondary education or life skills and vocational training that are available to other children are routinely refused to children with disabilities. The failing of educational systems to prepare youth with disabilities for the world of work, compounded by and contributing to their lack of skills, gives employers justification for discrimination against this group (Roggero et al. 2006).

Social security systems cause a further restraint to youth with disabilities becoming employed. Turton (2001) and Roessler (2002) described the discouraging effect of the UK and USA welfare benefit systems to people with disabilities. Recipients of benefits consider the risks associated with losing the benefit as too high should they become employed. They further consider the apparent cost of going to work as a deterrent to pursuing employment. Engelbrecht and Lorenzo (2010) described the same adverse effect of the social security grant, when employment is indeed an option for people with disabilities.

The low employment rate of youth with disabilities is further influenced by government policies that are not being implemented, or not being implemented effectively, along with market inefficiencies. The result is an imbalanced and out-of-sync supply and demand dynamic in labour markets (Roggero et al. 2006). Roulstone suggests that the changing nature of employment, global challenges for disabled workers, and the role of the state and trade unions need to be reconsidered in transforming the global capitalist economy (2002). Failing this, current labour markets will remain exclusionary to youth with disabilities, and continue to support a mainstream system of poverty and unemployment (Roulstone 2002).

Neoliberal workfare policies, where economic policy favours a movement from welfare to work, seem to have created tension between person-centred principles and the simultaneous improvement of service efficiencies and accountability. In three developed countries with healthy market economies where workfare policies have become operational, many people with disabilities remained unable to access the support they need to participate fully in the labour market (O’Brien & Dempsey 2004); this, despite the availability of employment strategies. In Finland, for example, sheltered employment^1^ remains the largest and most common employment option for people with disabilities, even though affirmative businesses are available to transition people with disabilities into real work. Sweden has subsidised employment (competitive employment with up to 80% wage subsidy to employers, or a job coach paid by the state), and in both countries, as well as in Australia, supported employment^2^ (SE) is available as a work transition strategy.

Evaluations of the effect of workfare policies on equality in employment participation of people with disabilities have shown only modest success (Harris, Owen & Gould 2012). In the USA, UK and Australia, researchers found that an individualised model of citizenship is promoted by these policies that systematically ignore the social, economic and labour market conditions in which individuals seek employment (Harris et al. 2012). The model has a further adverse impact on people who already experience high levels of discrimination in free markets, because services can operate selectively and become prone to serve those with a higher likelihood of entering competitive employment. This ‘individualisation of disability’ maintains the marginalisation of people with disabilities, when it is mainly political and organisational forces that create exclusive societies (Eide & Ingstad 2013:5).

Literature thus confirms that international attempts at social and economic policy levels to improve the employment situation for youth with disabilities render minimal outcomes at best, and are ineffective at worst. Evidence produced at levels where work transition occurs, will subsequently be examined to contribute to this comprehensive review of work transitions and youth with disabilities.

## Evidence about mechanisms for work transition programmes

Several studies describe the characteristics of programmes that are needed to transition people with disabilities into employment. Robinson (2000) and Smits (2004) identified collaboration and communication between agencies, having employment for people with disabilities as a shared priority, and service providers, public awareness and involved employers as central factors in employment inclusion. Smits, who researched best practice in disability employment in the USA, further found that positive employment outcomes were facilitated at service sites when services were integrated and coordinated with common, customer-driven objectives, and traditional bureaucratic barriers were avoided. At community level, the co-location of staff at employment service centres, and cross-training staff about each other’s roles, builds trust among providers and promotes collaboration. Accessibility and state-of-the-art assistive technology further maximises the value of services provided. Smits further emphasised the availability of multi-agency expertise to consumers, with shared accountability reinforcing the provision of high quality shared services (2004). Another study focused on assisting people with mental illness into employment, found that liaison positions and collaborative teams, staff training on mental health and workforce issues, and multi-level involvement of people with disabilities enhance successful work transitions (Boeltzig, Timmons & Marrone 2008).

In American literature, school-to-work programmes were overwhelmingly found to be effective in transitioning youth with disabilities into work. Rabren, Dunn and Chambers (2002) researched predictors of post-school employment for learners with disabilities, agreeing that positive employment outcomes can be expected from high school programmes that engage students in work (i.e. a focus on transition out of school into work). These programmes, (see the Youth Transition Program Model, Benz, Lindstrom & Latta 1999; the Transition Service Integration Model, Luecking & Certo 2003; Project SEARCH, O’Day 2009; Rutkowski et al. 2006; the Transition and Customised Employment Project, Rogers et al. 2008; the Partnerships for Youth Initiative, Muthumbi 2008 and the Youth Transition Demonstration Project, Luecking & Wittenburg 2009) usually involved collaborative efforts among a number of agencies (e.g. the Department of Education, state vocational rehabilitation agencies and employers), and interventions spanning the last year of a youth’s secondary schooling until sustainable employment had been secured.

Other mechanisms that have been found to be viable for transitioning youth with disabilities into employment are micro-enterprises and affirmative businesses. Conroy, Ferris and Irvine (2010) studied micro-enterprises in Alabama State (USA) and concluded that participation in employment is promoted for people with intellectual and developmental disabilities through this mechanism. In the USA and UK, affirmative businesses were found to be relevant as employment options for people with disabilities, especially where few competitive employment opportunities are available (Easterly & Mccallion 2010; Secker, Dass & Grove 2003).

Literature from industrialised countries overwhelmingly reflects positive employment outcomes for youth with disabilities when specific approaches are implemented at programmatic levels where transition occurs. To conclude this review though, the same or similar evidence from the developing world will now be considered to ascertain what the reality for youth with disabilities in this context may be.

## Evidence of work transitioning efforts in the developing world

Research from the Global South has commented on the very limited success of micro-financing as an employment strategy for people with disabilities. Lewis (2004) researched self-employment of women with disabilities in Zambia and Zimbabwe, when they made use of micro-financing. She concluded that key strategies still need to be put in place to include women with disabilities in finance, in order for micro-financing to be a viable transition strategy. De Klerk (2008) also found that this strategy is restricted for people with disabilities in other African countries, India and the Middle East, because of stigmatisation and self-exclusion. People with disabilities also do not have prior business experience, and micro-financers are often absent in rural areas (De Klerk 2008). Nuwagaba and Rule (2016) highlighted that people with disabilities in Uganda cannot access learning about micro-finance.

A South African study, conducted almost 20 years ago, found that home industries,^3^ as an employment option for people with disabilities in rural areas, were non-viable (Uys & Phillips 1997). Though small business- and institution-based approaches were successful in creating employment, the total number of people with disabilities who became employed was very low and, as such, the cost-effectiveness of the researched approach was questioned (Uys & Phillips 1997).

Some research has been conducted with large employers in South Africa, finding that employers are willing to employ people with disabilities, if certain conditions are met. Wiggett-Barnard and Swartz (2012) surveyed 26 large South African employers’ perspectives, and found that these employers are more prone to hire people with disabilities in the next 12 months if they had already hired people with disabilities before (*Z* = 5.45; *p* < 0.05). Employers identified the use of specialised recruitment agencies, a targeted recruitment plan, disability awareness training for staff and internships as the best facilitators for the employment of people with disabilities. Most participants also indicated that a special budget for accommodation would enhance facilitation of disability-employment. Participants valued information on accommodation and the impact of disabilities on job performance, leading the researchers to conclude that better information sharing and understanding can lead to better representation of people with disabilities in the South African labour market (Wiggett-Barnard & Swartz 2012).

Contrary to employers’ perspectives, Marsay (2014) explored the narratives of people with disabilities to identify ways of facilitating employment for them. Her participants identified policy, support structures, education and training, individual and societal attitude shifts, self-determination and enabling environments as crucial factors in the transitioning of people with disabilities into the South African labour market. Ned and Lorenzo (2016) contributed by highlighting the need for capacity development of community-based service providers in rural South Africa, to enhance economic inclusion of youth with disabilities.

Supported employment was identified as a viable strategy for transitioning people with disabilities into work in contexts with limited resources (Van Niekerk et al. 2015). Van Nierkerk et al.’s study followed people with mental disabilities over a period of 12 months and tracked their utilisation of supported employment services and employment outcomes for that period.

With the South African context in particular having distinguished itself in the available literature, the review progressed by sharply focusing on this local context, and how work transition programmes may be facilitating the participation of youth with disabilities in competitive employment.

## South Africa’s disability employment environment

Programmes in the public domain in South Africa continue to come up short on positive employment outcomes for youth with disabilities, confirming that there is a lack of policy implementation. South Africa signed and ratified the United Nations Convention on the Rights of People with Disabilities in 2007, pledging to protect the right of people with disabilities to work on an equal basis with others, including the opportunity to gain a living by work that is freely chosen or accepted in a labour market that is open, inclusive and accessible (United Nations 2016). The National Planning Commission also specifically recognises the need for better reflection of people with disabilities in all levels of employment by 2030 (National Planning Commission 2012). Despite having this policy environment that is supportive of youth, South African youth with disabilities navigate poor health and social attitudes in their quest to become employed in addition to lack of skills and availability of jobs (Cramm et al. 2013).

In terms of policy implementation, there is no evidence available on whether the Department of Health’s strategy to assist people with disabilities into work, that is, vocational rehabilitation (VR), is effective. Health service consumers have access to VR services which include work assessment and preparation, but do not extend to transition into work (Coetzee et al. 2011).

For schoolgoing youth with disabilities, preparation for the world of work is insufficient, resulting in the low probability of successful transitioning into employment. Although the Department of Education offers a special education curriculum in schools for learners with special educational needs (LSEN), vocational training as a channel for work transition is not a focus of this curriculum (Steyn & Vlachos 2011). As such, career services in special needs education remain very limited. Though a number of mechanisms exist to assist youth with disabilities into tertiary education, the scope of this review excluded studies on the section of the disabled South African population who would typically be in a position to utilise such opportunities.

The National Skills Development Strategy (NSDS), implemented jointly by the Department of Labour and Department of Education, performs weakest on its equity targets for people with disabilities (Akoojee, Gewer & Mcgrath 2005). Enrolment of people with disabilities in NSDS skills development programmes has been extremely low (less than 1%) (HSRC 2009), with no specifics being reported about the enrolment and outcomes for youth with disabilities. The extent to which the NSDS has facilitated unemployed people into employment has also been restricted by high levels of poverty and unemployment (Kay & Fretwell 2003).

The National Department of Public Works (NDPW) failed to create employment for youth with disabilities through their Expanded Public Work Programme (EPWP). The EPWP entails the use of public expenditure to promote productive employment and develop marketable skills among historically disadvantaged communities (International Labour Office 2014). By 2014, between 0.001% and 0.003% people with disabilities had become employed through EPWP, despite a target of 2% of 4.5 million people (Department of Public Works & South African Cities Network 2014). Specific information on the number of youth with disabilities was again not reported.

Though the state is trying to honour its commitment to the United Nations Convention on the Rights of People with Disabilities by recognising the plight of youth with disabilities in legislation and policy, implementation of policy in state departments has failed to change the employment situation of youth with disabilities.

## Limitations of the literature review

Although a number of initiatives exist under the *Broad-based Black Economic Empowerment Act* of 2003, and the *Preferential Procurement Policy Framework Act* of 2000, the scope of this review excludes self-employment and industry ownership initiatives facilitated by these Acts. A separate and growing body of knowledge exists about entrepreneurship as a strategy for people with disabilities to become economically active.

Tertiary education options and mechanisms to assist youth with disabilities who may have obtained further education after school were also not considered in the scope of this article. This review focused on the largest portion of youth with disabilities who generally would not be able to access further education.

Specific social issues that may intersect with the concepts of youth and disability were not included in the scope of this review.

## Conclusion

Because of the lack of enforcement of disability supportive laws and failure to implement related policies, youth with disabilities remain marginalised and excluded from a job market that is saturated with an over-supply of unskilled workers. Although South Africa’s policy environment supports the right of youth with disabilities to work and highlights access to employment for this group as a priority, youth with disabilities continue to lose out on employment against other designated groups defined by the law.

The lack of evidence regarding the employment of youth with disabilities has resulted in a shortfall in the design of measures that will effectively address their employment needs. In the developed world, work transition programmes for youth with disabilities have been met with varying degrees of success through integrated school-to-work approaches. This appraisal of the international literature concluded though that the majority of reported studies were focused on disability employment in general, with less attention to youth with disabilities. In developing countries, available research on employment outcomes for this sub-group is even more limited or altogether absent. Efforts to assist youth with disabilities into employment will continue to be inadequate, if specific evidence-based transitioning methods and avenues are not identified and researched.

Research of policy implementation becomes all the more important when evidence can inform the development of effective delivery mechanisms of work transition for youth with disabilities. The redress of past and current injustices in the employment of youth with disabilities shall ultimately have a positive influence on the unemployment rate of this group. It is well known that the financial reward from participating in work is only one of a range of benefits to the worker, including social contacts and support, and the structuring of time (Boland & Griffin 2015; Van Niekerk 2009; Webster & Omar 2003). In the absence of effective mechanisms to transition youth with disabilities into employment, these health-giving elements of work remain exclusive to those without disabilities, and youth with disabilities stay on the margins of society, unable to participate.

## Footnotes

1 Sheltered employment refers to segregated work programmes for people with disabilities who are not able to work in a competitive employment setting (Krainski 2013).

2 Supported employment is an employment strategy that facilitates people with severe disabilities into competitive employment (Wehman et al. 1991).

3 Industries where workers performed piecework at their homes for local businesses.
